# A newly developed algorithm for switching outpatient medications to medications listed in the hospital formulary: a prospective real-word evaluation in patients admitted electively to hospital

**DOI:** 10.1007/s00228-024-03682-w

**Published:** 2024-04-24

**Authors:** Finja Möller, Malte Oetting, Andreas Spiegel, Olaf Zube, Thilo Bertsche

**Affiliations:** 1 Pharmacy Department, Bundeswehr Hospital Hamburg, Hamburg, Germany; 2 Central Clinical Management, Bundeswehr Hospital Hamburg, Hamburg, Germany; 3 Drug Safety Center, Leipzig University and Leipzig University Hospital, Leipzig, Germany; 4 Department of Clinical Pharmacy, Institute of Pharmacy, Medical Faculty, Leipzig University, Leipzig, Germany

**Keywords:** Medication review, Medication reconciliation, Outpatients, Hospitalization, Healthcare sector

## Abstract

**Purpose:**

In many countries, outpatient and inpatient care are separated. During hospitalization, therefore, switching the outpatient medication to medication of the hospital formulary is required.

**Methods:**

We newly designed a switching algorithm in six switching steps (S0–S5) and conducted a study at Bundeswehr Hospital Hamburg (300 beds, 80% civilians). We performed (i) a medication reconciliation to obtain information on outpatient medications and (ii) a medication review to solve drug-related-problems, e.g., drug-drug interactions. We applied (iii) the algorithm to switch medications to the hospital formulary.

**Results:**

(i) We identified 475 outpatient medications (median per patient: 4; Q25/Q75 2/7) in 100 patients consecutively admitted to hospital (median age: 71; Q25/Q75: 64/80 years). Of 475 medications, the switching algorithm could not be used since product names were missing in 23.9% and strength in 1.7%. In 3.2%, switching was not required since medication was not prescribed during the hospital stay. (ii) Drug-drug interactions were identified in 31 of 79 patients with more than one medication. (iii) Of 475 medications, 18.5% were on the hospital formulary and therefore did not need to be  switched (S0), 0.2% were on a substitution-exclusion list not allowing switching (S1), 42.0% were switched to a generic medication of the hospital formulary (S2), 1.7% to a therapeutically equivalent medication (S3), 0.4% were patient-individually switched (S4), and for 8.2% a standardized/patient-individual switching was not possible (S5).

**Conclusions:**

Despite comprehensive medication reconciliation, patient- and medication-related information for switching medications to the hospital formulary was often missing. Once all the necessary information was available, standardized switching could be easily carried out according to a newly developed switching algorithm.

## Introduction

In most countries, outpatient and inpatient sectors of the healthcare systems are clearly separated [1–4]. This also applies to the reimbursement [1–4]. In contrast to outpatients, the supply of medications for inpatients is mainly provided by hospital pharmacies [5]. According to the legal provisions of many countries, hospital pharmacies may only supply medicines for the care of patients undergoing inpatient treatment or for direct use in approved outpatient departments [5].

The costs of inpatient medicines are covered by a flat rate system (i.e., diagnoses-related groups) and are not usually reimbursed separately by healthcare insurances. The costs of inpatient medicines are regularly lower than in outpatients, e.g., due to direct procurement from the pharmaceutical industry. As a consequence, for economic reasons a hospital formulary does not cover the entire spectrum of the pharmaceutical market as this is the case for outpatients [5]. Therefore, medication switching has to be performed at the beginning of a hospital stay. These switches are not medically necessary, but merely result from economic requirements.

Outpatient medications are frequently prescribed by various physicians, such as general practitioners and specialists. This provides a high potential of drug-related problems such as drug-drug interactions and unintended medication modifications [6–8]. One positive result from switching outpatient medications during hospital admission might be that drug-drug interactions, for example, are recognized and resolved when a medication review is concurrently performed. In order to perform a switch and a medication review in a sophisticated manner, all relevant patient- and medication-related information should be collected in a medication reconciliation.

The attending hospital physicians are responsible for the accuracy and appropriateness of the entire medication regimen for their inpatients. Thus, a highly specialized physician in the hospital may have to assume overall responsibility for the whole medication regime including medications from other specialists. The hospital physician may therefore be inclined to request outpatient medication on the basis of individual orders instead of switching them to the hospital formulary. This would lead to comparatively high costs for the pharmacy, as individual orders have to be placed with wholesalers.

Therefore, solution strategies such as standardization through the use of switching algorithms and the involvement of pharmacists have already been investigated as recently reported [9–11]. However, the legal standardization of medication lists, e.g., through the Federal Medication List in Germany [12, 13], should have significantly changed the framework conditions to obtain all relevant patient- and medication-related information for switching in recent times. Bottlenecks in the availability of medications have recently made switching even more complex [14, 15]. This is because rapid adjustments to the respective delivery situation are becoming increasingly important. This applies also to the medication switching at interfaces. As a consequence, the hospital formulary is constantly changing—even at short notice.

In summary, all these changes in the healthcare system make it sensible to critically analyze the switching of medication during hospital admission in the context of the current situation in healthcare. Additionally, known strategies are to be further designed in line with current circumstances. In this way, also hospitals outside of the universities’ medical care system should be considered.

Within this study, we therefore newly designed a switching algorithm considering current requirements. We then applied it to real patients admitted to hospital in a non-university setting. In this way, we aimed to investigate to what extent the information on outpatient medications required for switching to the hospital formulary would be available under routine conditions. Additionally, we aimed to explore to what extent the algorithm would be appropriate to standardize the switching process. By this, not standardized individual switching and orders with wholesalers should be decreased to a minimum.

## Methods

### Participants and setting

Elective patients consecutively admitted to our Urology, Otolaryngology, Oral and Maxillofacial Surgery, General and Visceral Surgery, and Oncology Department were invited to participate. The study was performed during a 3.5-month period from January 2, 2023, to April 14, 2023, in a 300-bed military hospital (Bundeswehr Hospital Hamburg). The hospital offers tertiary care for civilian and military patients. As the hospital is integrated into the public health and emergency services, 80% of the beds are available for civilian patients and 20% for military patients. The basis for every inpatient medication treatment is a hospital formulary. It contains all the product names listed for use in the hospital. The list has been compiled by the responsible Drug Commission, particularly at the request of the respective healthcare institutions. On admission to hospital, outpatient medications have to be switched to the medication listed on the hospital formulary. Before the study was performed, this was carried out by the wards and, if necessary, the pharmacy was asked for recommendations in individual inquiries.

### Study design

The study was divided into the following parts.

1. Switching algorithm
  An expert panel of physicians and pharmacists (i.e., authors of this article) designed an algorithm with six consecutive switching steps from S0 to S5. The algorithm was taking into account current internal and legal requirements with the aim of enabling a standardized switching for outpatient medication to the medication listed in the hospital formulary.
2. Study execution
  We consecutively invited electively admitted patients to give their consent before participating.
  2.1 ***Medication reconciliation including patient interview: ***We performed a medication reconciliation including a patient interview offered by a pharmacist. In addition, the clinical information system was searched for relevant patient- and medication-related information (e.g., previous physician’s letters, discharge letters). By this, we aimed to obtain a comprehensive database to perform a medication review and to apply the switching algorithm.
  2.2 ***Medication review:*** A pharmacist performed a medication review in the enrolled patients based on the patient- and medication-related information from the medication reconciliation. By this, we aimed to solve drug-related problems before applying the switching algorithm.
  2.3 ***Algorithm-based outpatient medication switching:*** A pharmacist prepared a switching list for the respective wards, which could be used to perform the switch on the ward or to order individual products. By this, we aimed to switch the outpatient medications to medications listed in the hospital formulary as far as possible or to prepare an order of the outpatient medication with the wholesalers.

### Ethical approval

The Medical Faculty of the University of Leipzig (#183/22ek of 24 May 2022) and the research committee of the Bundeswehr Hospital Hamburg both granted ethical approval for the project.

### Switching algorithm

The switching algorithm was designed on the basis of the switching experience in the setting and international literature [9–11] by a panel of medical and pharmaceutical experts (consisting of the authors of this article).

Combination products that were switched to mono-products (e.g., generics) were counted as generic switching. In the case of combination products containing several active ingredients, the original combination product was counted as one switch.

The algorithm was pre-tested in advance on sample medications from patients who were independent from the main study.

**Fig. 1:**
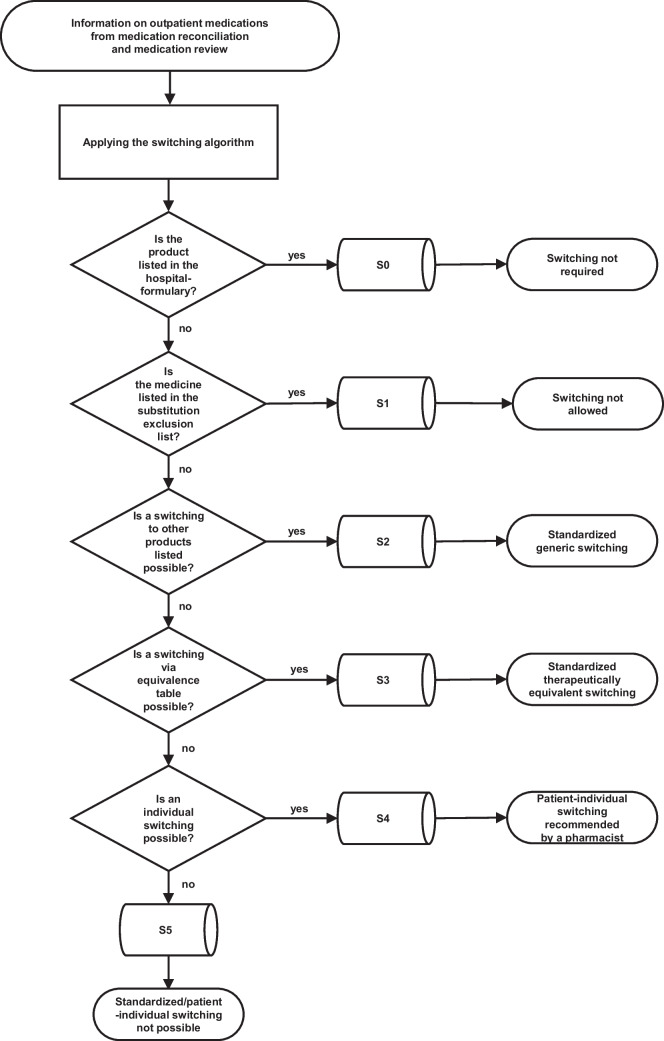
A new switching algorithm designed by an expert panel of physicians and pharmacists

The switching algorithm was divided into several consecutive switching steps, which had to be executed one after the other according to the algorithm shown in Fig. 1. The algorithm consisted of the following six consecutive switching steps, ranging from S0 to S5. We only switched within the respective dosage form.

- *S0 (switching not required):* no switching of the medication was required since the product was listed in the hospital formulary and could be ordered from pharmacy stock.
- *S1 (switching not allowed):* no switching of the medication was allowed since specific pharmacodynamic or pharmacokinetic properties were associated with risks for the patient if switched. For this purpose, the following active ingredients were determined by the responsible Drug Commission not to be switched to other products based on information from [16]: carbamazepine, phenobarbital, phenytoin, primidone, valproic acid, ciclosporin, tacrolimus. It was recommended that the medication should be ordered on special request, signed by a senior physician.
- *S2 (standardized generic switching, “aut idem”):* switching was recommended since the same active ingredient and dose but different product (e.g., generics) were available in the hospital formulary. An alternative product from the hospital formulary was recommended to be ordered from the pharmacy stock.
- *S3 (standardized therapeutically equivalent switching, “aut simile”):* switching was recommended within the corresponding ATC Level 5 (in exception Level 4; e.g., from simvastatin to rosuvastatin or according to a specific insulin switching table) and exclusively considering defined equivalent doses (switching only recommended if explicitly stated on an equivalence table). Equivalence dose tables from the Federal Union of ABDA-German Associations of Pharmacists were considered for this purpose [17]. Here, it was determined which substances may be switched to others and which may not. Equivalent dosages from those tables were considered if appropriate. The alternative medication listed in the table and being listed in the hospital formulary was then recommended to be ordered from the pharmacy stock.
- *S4 (patient-individual switching recommended by a pharmacist):* switching was recommended from one medication group to another (e.g., alpha-1 blocker for lowering blood pressure to beta-blocker) in an individual switching after careful consideration by a pharmacist for the individual patient. The alternative medication was then recommended to be ordered from the pharmacy stock.
- *S5 (standardized/patient-individual switching not possible):* if all other switching steps did not lead to a recommendation, an order from the wholesaler was prepared.

### Medication reconciliation including patient interview

The participating wards were invited to hand out an information brochure about the project when appointments were made for elective patients. By this, patients were invited to voluntarily participate in the study. We then recorded all available patient-related medication information, in particular from the clinic information system and archived physician’s letters, in a medication reconciliation. In addition, a patient interview with a pharmacist was performed during admission to the hospital. By this, additional information should be obtained directly from the patient (and the patient’s relatives, if applicable). When making the appointment for the hospital admission, patients were also asked to bring all their medications for a brown bag review. Medication lists (e.g., the Federal Medication List as it has to be issued by the general practitioner in Germany). Other medication records such as physician’s letters were also considered.

### Medication review

Before the switching algorithm was applied, drug-related problems were identified and recommendations to solve them were forwarded to the attending physicians if applicable. As part of this, drug-related problems were analyzed (e.g., drug-drug interactions, appropriateness of medication [18, 19]). For the latter, the PRISCUS [20] and FORTA list [21] were used to assess patient-related information.

### Algorithm-based outpatient medication switching

In order to carry out switching of the outpatient medication to the medications listed in the hospital formulary, the following information on the individual medications prescribed for outpatients was required: active ingredient(s), product name, strength, and times of administration. By using the switching algorithm, an order list was prepared by the pharmacist. Afterwards, the attending physicians were recommended to forward the prepared order list to the pharmacy. If they pass on the completed list, the pharmacy stock delivered the product (if listed in the hospital formulary) or it has to be ordered from the wholesaler’s (if not listed).

### Statistics and data evaluation

We reported numbers of patients in absolute numbers and in percentage in the above mentioned pre-defined switching steps (S0–S5). A descriptive statistical analysis was carried out for respective items. We present data as median (Q50) including the 25% (Q25) and 75% quartiles (Q75) as appropriate.

### Study size

Based on previous work, considering the heterogeneity of the admission departments and our experience with similar studies in the same setting [9–11], we assumed a patient number of at least 100 patients to be sufficient to draw conclusions in our setting and to find a pragmatic approach that can be realized in the time available.

## Results

### Patient characteristics

In this study, 100 patients with a median age of 71 years (Q25/Q75: 64/80 years) were enrolled. The enrolled patients were treated with a total of 475 medications (median 4; Q25/Q75: 2/7 per patient) with 511 active ingredients before admission. The patients were mainly assigned to urology in 87% (87/100) and to a lower extent also to general surgery in 8% (8/100), to trauma surgery in 4% (4/100), and to internal medicine in 1% (1/100).

### Medication reconciliation including patient interview

For 21% (21/100) of patients, the main source of information was the medication lists brought in. For 15% (15/100), the main source of information was medical documents such as the physician’s letter and for 64% (64/100) patient’s information reported from the interview.

For 23.9% (114/475) of the total of 100 patients with 475 medications, the product name was not known. For 1.7% (8/475), the strength of the outpatient medication was unknown. Of the total of 475 medications, 3.2% (15/475) did not have to be switched because those medications were not prescribed during the hospital stay. For example, they were discontinued or paused as well as given only sporadically, possibly according to a special therapy regime (as in oncology).

### Medication review

#### Drug-drug interactions

For 79% (79/100) of all patients with at least two concomitant medications, it was possible to assess drug-drug interactions between the outpatient medications. In 39% (31/79), at least one drug-drug interaction was identified. The most common active ingredients involved in the drug-drug interactions were acetylsalicylic acid and amlodipine, each with 15 respective drug-drug interactions. Therapy was actually optimized for one medication: omeprazole was replaced with pantoprazole due to its drug-drug interaction potential with clopidogrel.

#### Potentially inappropriate medications

In 16% (12/73) of patients being 65 years or older, at least one potentially inappropriate medication was identified. In total, 3 active ingredients were found on the PRISCUS list and 12 active ingredients that were divided into categories C and D on the FORTA list. In none of these cases, a treatment adjustment was considered necessary.

### Algorithm-based outpatient medication switching

#### Application of the switching algorithm in general

**Fig. 2:**
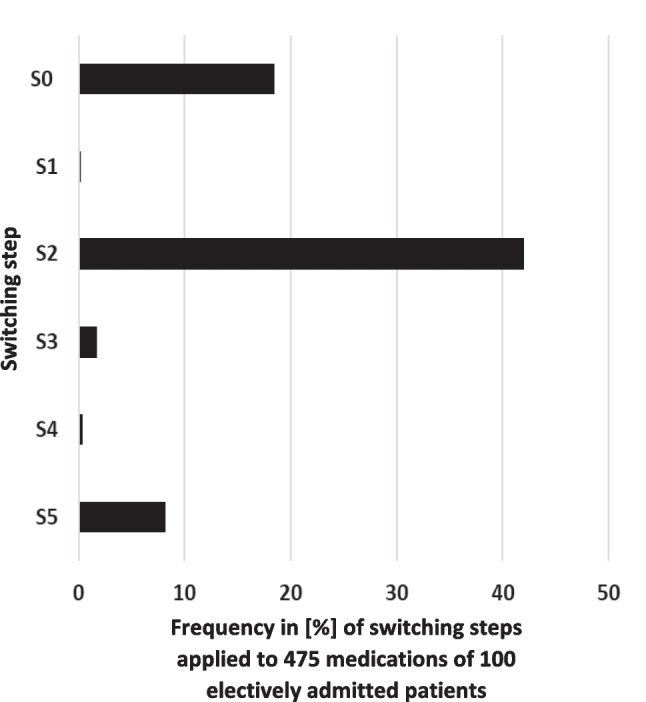
Frequency in [%] of switching steps applied to 475 medications of 100 electively admitted patients: S0 (switching not required), S1 (switching not allowed), S2 (standardized generic switching, “aut idem”), S3 (standardized therapeutically equivalent switching, “aut simile”), S4 (patient-individual switching recommended by a pharmacist), and S5 (standardized/patient-individual switching not possible). It was not possible to apply the switching algorithm in 23.9% (114/475) since the name of the product was unknown and in 1.7% (8/475) since the strength of the drug was unknown. 3.2% (15/475) did not have to be switched because those medications were not prescribed during the hospital stay

The proportions of the recommended switching steps according to the algorithm are shown in Fig. 2. In relation to all 100 participating patients with a total of 475 medications, 18.5% (88/475) of the outpatient medications were listed in the hospital formulary, i.e., were assigned to switching step S0 and could be obtained from the pharmacy stock. 0.2% (1/475) of the medicines were assigned to switching step S1 indicating that switching was not allowed. At 42.0% (200/475), switching step S2 was applied as a standardized generic switching. 1.7% (8/475) were assigned to switching step S3 as a standardized therapeutically equivalent switching. 0.4% (2/475) were assigned to switching step S4 consisting of a patient-individual switching recommended by a pharmacist. 8.2% (39/475) of the medicines were assigned to switching step S5 including all medications for which a standardized or patient-individualized switching was not possible.

#### Application of the switching algorithm to combination products

In a total of 23 products, the most common combination products consisted of hydrochlorothiazide in combination with candesartan or an ACE inhibitor (30%, 7/23) followed by the combination of ezetimibe with a statin (13%, 3/23) and metformin with a DPP4 inhibitor (13%, 3/23). The combination of dutasteride with tamsulosin occurred in 9% (2/23). Of all those 23 combination products, 39% (9/23) were switched to a combination product and 57% (13/23) to a mono-product. 17% (4/23) required no switching, i.e., were classified in switching step S0. None (0%, 0/23) was classified in switching step S1, 52% (12/23) in switching step S2, 4% (1/23) each in switching step S3 and S4, and 17% (4/23) in switching step S5.

#### Application of the switching algorithm to defined medications

The individual medications identified through the medication reconciliation and their switching are listed in Table 1. The three most frequently identified medications for applying the switching algorithm were acetylsalicylic acid 6.1% (29/475), tamsulosin 5.7% (27/475), and amlodipine 4.4% (21/475). There were no dose equivalence tables for two specific groups, so an individual order was unavoidable according to switching step S5. The first group comprises urologicals (ATC code G04), for which the active ingredients solifenacin, desfesoterodine, oxybutynin, silodosin, and alfuzosin were each ordered once. The second group relates to antidiabetics with the ATC code A10B. Three times, a GLP1-analogon was ordered and once each a thiazolidinedione (pioglitazone) and a DPP4 inhibitor (vildagliptin).

**Table 1:** Medications identified in a total of 475 medications in 100 enrolled patients for the application of a standardized switching algorithm consisting of the following switching steps: S0 (switching not required), S1 (switching not allowed), S2 (standardized generic switching, “aut idem”), S3 (standardized therapeutic equivalent switching, “aut simile”), S4 (patient-individual switching recommended by a pharmacist), and S5 (standardized/patient-individual switching not possible)

**#**	**Active ingredient**	**Relative frequency [%]**	**Absolute frequency** **[*n*]**	**Absolute** **numbers** **[***n***]** **(switching** **step)**
**1**	Acetylsalicylic acid	6.1	29/475	*n* = 3 (S0^a^); *n* = 11 (S2^b^); *n* = 2 not to be switched as not prescribed during the hospital stay; *n* = 13 product not known
**2**	Tamsulosin	5.7	27/475	*n* = 6 (S0^a^); *n* = 11 (S2^b^); *n* = 10 product not known
**3**	Amlodipine	4.4	21/475	*n* = 2 (S0^a^); *n* = 12 (S2^b^); *n* = 7 product not known
**4**	Metoprolol (succinate or tartrate)	4.0	19/475	*n* = 7 (S0^a,c^); *n* = 6 (S2^a,c^); *n* = 2 (S5^d^); *n* = 3 product not known; *n* = 1 dosage not known
**5**	Atorvastatin	3.8	18/475	*n* = 9 (S2^e^); *n* = 8 product not known; *n* = 1 dosage not known
**6**	Ramipril	3.6	17/475	*n* = 8 (S2^e^); *n* = 9 product not known
**7**	L-thyroxine	3.4	16/475	*n* = 2 (S0^a^); *n* = 7 (S2^b^); *n* = 6 product not known; *n* = 1 not to be switched as not prescribed during the hospital stay
**8**	Colecalciferol	3.2	15/475	*n* = 8 (S0^a^); *n* = 7 (S2^b^)
	Simvastatin	3.2	15/475	*n* = 1 (S0^a^); *n* = 8 (S2^b^); *n* = 6 product not known
	Torasemide	3.2	15/475	*n* = 1 (S0^a^); *n* = 6 (S2^b^); *n* = 7 product not known; *n* = 1 dosage not known
**9**	Candesartan	2.9	14/475	*n* = 1 (S0^a^); *n* = 6 (S2^b^); *n* = 7 product not known
	Dipyrone	2.9	14/475	*n* = 12 (S2^e^); *n* = 2 product not known
	Pantoprazole	2.9	14/475	*n* = 1 (S0^a^); *n* = 10 (S2^b^); *n* = 3 product not known
**10**	Metformin	2.5	12/475	*n* = 8 (S2^e^); *n* = 4 product not known
	Bisoprolol	2.5	12/475	*n* = 2 (S2^e^); *n* = 7 product not known
**11**	Rosuvastatin	2.3	11/475	*n* = 1 (S0^a^); *n* = 10 (S2^b^)
**12**	Allopurinol	2.1	10/475	*n* = 5 (S2^b^), *n* = 4 product not known; *n* = 1 dosage not known
**13**	Ibuprofen	1.9	9/475	*n* = 1 (S0^f^); *n* = 8 product not known
**14**	Apixaban	1.7	8/475	*n* = 8 (S0^f^)
	Rivaroxaban	1.7	8/475	*n* = 8 (S0^f^)
**15**	Omeprazole	1.5	7/475	*n* = 6 (S3^g^); *n* = 1 (S4)
	Hydrochlorothiazide	1.5	7/475	*n* = 1 (S0^a^); *n* = 6 (S2^b^)
**16**	Salbutamol (albuterol)	1.3	6/475	*n* = 1 (S0^a^); *n* = 5 (S2^b^)
**17**	Insulin glargine	1.1	5/475	*n* = 3 (S0^a^); *n* = 2 (S2^b^)
	Empagliflozin	1.1	5/475	*n* = 5 (S0^f^)

^a^If the specific product of the enrolled patient was listed in the hospital-formulary

^b^If the specific product of the enrolled patient was NOT listed in the hospital formulary

^c^For metoprolol succinate (one product on the inhouse list) only

^d^For metoprolol tartrate (NO product listed in the hospital formulary) only

^e^Specific products of the enrolled patients were switched to a generic product listed in the hospital formulary

^f^Specific products of the enrolled patients were listed in the hospital formulary

^g^Specific products of omeprazole were switched to pantoprazole listed in the hospital formulary

## Discussion

### General considerations

In our study, we enrolled 100 patients electively admitted to hospital. We investigated the application of a switching algorithm designed according to current requirements. This algorithm was divided into six successive steps. To apply the switching algorithm in routine care to an outpatient medication, we, first, collected all relevant information on outpatient medications via a medication reconciliation. The patient interview by the pharmacist proved to be the richest source for compiling the required information. The algorithm, however, could not be used for around a quarter of the identified medications due to missing patient-related information on the respective outpatient medications.

Before applying the switching algorithm, second, a medication review conducted by a pharmacist identified and resolved several drug-related problems. We focused on drug-drug interactions and potentially inappropriate medications while assessing drug-related problems. Of the 10 most frequently prescribed outpatient medications, only 2 were not listed in the hospital formulary. For those products which were not listed in the hospital formulary, most switches could be performed by using the standardized algorithm, with generic switching being the most common. Only rarely, an individual decision had to be made if no standardized switching to the hospital formulary was possible after the previous steps of the algorithm.

Since recommendations were issued to the wards as an order template, switching recommendations within this project were actually implemented in routine practice.

### Medication reconciliation including a patient interview

For around a quarter, the information regarding product name and strength was not complete in our study. Therefore, switching could not be performed on the basis of the algorithm. In this case, time-consuming research would have been necessary to complete the patient- and medication-related information, or the concerning medication would have had to be discontinued during hospital stay without therapeutic justification. This shows that a consideration of the switching algorithm alone is not sufficient. The completeness of the required patient- and medication-related information should also be the subject of investigations. In this context, the patient interview by pharmacists contributed significantly to information completion according to our data.

We enrolled patients who were mainly admitted to the Urology Department. This indicates, at first glance, a fairly specific patient collective. At second glance, as the average age shows, we enrolled patients at an advanced age. It was, therefore, likely that our patients were additionally affected by several diseases of internal medicine. The top list of medications supports this assumption and nevertheless makes the results generalizable to other patients of elective hospital admission.

The pharmacist-led patient interview proved to be helpful in recognizing the requested information needs. In 64% of the patients, the drug information required for switching was compiled mainly by the patient interview.

Our results regarding the positive evaluation of the pharmaceutical patient interview are confirmed in the literature: For example, as reported in [22], the recording of medication use by means of patient interviews appears to be essential in identifying drug-related problems. By this, also self-medications can be considered for switching in principle. As a principle rule, however, we did not transfer self-medication to the hospital formulary or ordered those medications. In this context, we defined self-medication in the sense that a medication was taken by patients themselves without a physician’s prescription. Patients were instructed not to self-medicate during their hospital stay. Non-prescription medications, in contrast, which were prescribed by a physician, were switched.

### Medication review

The evidence for medication reviews in the literature is very clear, as a recent Cochrane review [23] underlines. In adult hospitalized patients, medication reviews probably decrease the number of hospital readmissions and emergency department visits. Little to no effect was shown, however, on mortality [23]. The effect on health-related quality of life is described as very uncertain [23]. This is confirmed by another review [24] reporting that an isolated medication review during a short intervention period had effects on most medication-related outcomes. In contrast, minimal effects on clinical outcomes and no effects on quality of life were described by the same authors [24].

In our study, performing a medication review before applying the switching algorithm could be an essential step in optimizing drug safety. In our study, we have shown that drug-drug interactions occurred in 39% of those 79 patients who had more than one continuous medication. Potentially inappropriate medications (PIMs) were found in 16% of those 73 patients who were 65 years or older. However, in this study, we found that only one recommendation actually brought about a change—i.e., avoidance of drug-drug interactions concerning omeprazole by switching to pantoprazole.

Although various professional groups as well as nursing services have been involved in medication reviews [25], the involvement of a pharmacist in medication reviews in particular is frequently mentioned in the literature [26]. However, the focus here is more on discharge [27] and measures to reduce (re-)admission to hospital [28]. With regard to interfaces, medication reviews in outpatient care [29] and offered by community pharmacies [30] have also been examined. However, reviews on admission as reported by us in this work are comparatively rare, especially when they are associated with a consecutive medication switching. Although it can certainly be assumed that this is a routine measure in many hospitals, the number of published scientific studies as ours is comparatively low.

### Algorithm-based outpatient medication switching

So far, the literature has mainly reported on therapeutically induced medication switching [31]. In recent years, however, quality assurance measures such as standardized switching by pharmacists and electronic prescribing aids have been implemented for switching induced through legal and economic reasons. Those strategies, however, were mainly implemented in university hospital settings. Therefore, we performed our study in a non-university setting addressing a standardized quality-assured switching. By this, we aimed to decrease follow-up costs due to a reduced number of expensive individual prescriptions.

An algorithm similar to that used in our study was applied to the medication schedules of 120 patients [9]. In this study, 774 medications prescribed at the time of their admission to a surgical department were assessed. However, in contrast to our study, which was conducted prospectively, the study in [9] was conducted retrospectively. We also included a wider range of electively admitted patients with a focus on urology. Furthermore, our study did not take place in a university hospital, which is often subject to special conditions in the context of maximum care that are not always transferable to other hospitals. In [9], 52.8% of the prescribed medications were included in the hospital formulary so that switching was not necessary. In contrast, only 18.5% were included in our hospital formulary. This shows the considerable difference of our data to a university hospital with a much more comprehensive hospital formulary.

In [9], 84.7% of the remaining medications were successfully switched to a corresponding generic medication and 10.2% to a therapeutic equivalent. In our study, 42.0% were a generic switching, while switching to a therapeutic alternative was recommended in 1.7% only. Even if the design of the algorithm cannot be compared one-to-one, the relative orders are largely consistent indicating that generic switching is usually possible and switching to a therapeutically equivalent is in comparison only rarely required.

The authors in [9] reported that for only 2.3% of the active substances no specific switching procedures were found according to the algorithm. In those cases, the medications were either discontinued or specific medication classes, current illnesses, or co-medication required manual switching as reported by the authors in [9]. What is more, only some medications were continued unswitched and ordered with a wholesaler. In our study, only for 0.4% a patient-individual switching had to be recommended by the pharmacist. Finally, 8.2% of outpatient medications still had to be ordered with wholesalers in our study.

It is also interesting to note that we succeeded in switching a total of 23 combination products in our study. Such products are very common in the outpatient sector and are intended to optimize adherence due to the small number of medications. Among the ten most frequently prescribed outpatient medicines, there were only two that were not included in the corresponding form listed in the hospital formulary. This shows that the significance of switching in the context of combination products is currently limited and that a simple switching to the hospital formulary is usually not a problem.

### Limitations

The algorithm was designed to structure and standardize the frequently rather subjective switching on the ward. In this way, the bias in this area decreased by the study concept in principle. A bias in this study may nevertheless lie in the fact that this study focused on electively admitted patients. In particular, the data basis from the study was comparatively better than for non-elective admissions, for example, admitted to the central emergency department. The following additional limitations have to be considered while drawing conclusions for an international setting from our results: In many patients, the patient- and medication-related information for a systematic switching according to the algorithm was not available in spite of a sophisticated medication reconciliation. Our non-university inhouse setting was a very specific hospital with apparently a very short list of available medicines in the hospital formulary. Last but not least, the general setting in Germany is a specific healthcare system driven by rebate contracts and strictly separate outpatient and inpatient sectors.

## Conclusion

The switch to the hospital formulary is an essential step in a quality-controlled medication process. As we have shown here, the majority of the medicines for switching were available in the hospital formulary. The others could mostly be switched generically to other product names mentioned in the hospital formulary. Medications that could not or were not allowed to be switched were comparatively rare. In addition to the algorithm, equivalence tables provided good service in switching. At the end, only a few medicines had to be switched individually by pharmacists or ultimately ordered from wholesalers. The main contribution of the pharmacist was to obtain a complete picture of the medicines to be switched during the patient interview. Better preparation prior to admission, such as instructions to bring all medication for a brown bag review, will increase the success of such measures in the future. A standardized algorithm can—as a future benefit of the results shown here best implemented in an electronic prescription tool—also simplify switching back from the hospital formulary to outpatient medication, e.g., as a medication recommendation in the discharge letter.

## Data Availability

The datasets generated during and analyzed during the current study are available from the corresponding author on reasonable request.
